# Role of age and health in perceptions of returning to work: a qualitative study

**DOI:** 10.1186/s12889-019-6819-9

**Published:** 2019-05-02

**Authors:** Joanne Neary, Srinivasa Vittal Katikireddi, Judith Brown, Ewan B. Macdonald, Hilary Thomson

**Affiliations:** 1 General Practice and Primary Care, Institute of Health and Wellbeing, University of Glasgow, 1 Horselethill Road, G12 9LX Glasgow, Scotland; 2 MRC Social and Public Health Sciences Unit, Institute of Health and Wellbeing, University of Glasgow, Glasgow, Scotland; 3 Public Health, Institute of Health and Wellbeing, University of Glasgow, Glasgow, Scotland

**Keywords:** Qualitative research, Unemployment, Older workers, Treatment burden, Return-to-work, Pre-retirement

## Abstract

**Background:**

People aged over 50 years form a growing proportion of the working age population, but are at increased risk of unemployment compared to other age groups. It is often difficult to return to work after unemployment, particularly for those with health issues. In this paper, we explored the perceptions, attitudes, and experiences of returning to work after a period of unemployment (hereafter RTW) barriers among unemployed adults aged over 50 years.

**Method:**

In-depth semi-structured interviews were conducted with a diverse sample of 26 unemployed individuals aged 50–64 years who were engaged with the UK Government’s Work Programme. Data were thematically analysed.

**Results:**

Age alone was not discussed by participants as a barrier to work; rather their discussions of barriers to work focused on the ways in which age influenced other issues in their lives. For participants reporting chronic health conditions, or disabilities, there was a concern about being unfit to return to their previous employment area, and therefore having to “start again” in a new career, with associated concerns about their health status and managing their treatment burden. Some participants also reported experiencing either direct or indirect ageism (including related to their health status or need to access healthcare) when looking for work. Other issues facing older people included wider socio-political changes, such as the increased pension age, were felt to be unfair in many ways and contradicted existing expectations of social roles (such as acting as a carer for other family members).

**Conclusion:**

Over-50s experienced multiple and interacting issues, at both the individual and societal level, that created RTW barriers. There is a need for employability interventions that focus on supporting the over-50s who have fallen out of the labour market to take a holistic approach, working across healthcare, employability and the local labour market, providing treatment and skills training for both those out of work and for employers, in order to create an intervention that that helps achieve RTW and its associated health benefit.

**Electronic supplementary material:**

The online version of this article (10.1186/s12889-019-6819-9) contains supplementary material, which is available to authorized users.

## Key points

- The current study conducted in-depth interviews with 26 unemployed over-50s to better understand their experience of barriers to RTW
- Barriers to work were defined as the consequence of the interplay between issues in different elements of participants lives
- Age was rarely mentioned as a barrier to work on its own. Rather, age was seen as an underlying factor which influenced other issues including health, health management, and access to the job market
- Interventions to support older workers returning to employment should focus on holistic joined-up working, looking to healthcare, as well as skills and employability services to enable RTW

## Background

Globally, human populations are ageing, with all OECD countries experiencing a steep increase in the proportion of older people in their populations, while witnessing a decrease in younger people [1]. Explanations of this phenomenon are linked with decreased fertility, improvements in medicine, and improved health behaviours. This demographic shift has also impacted on the average age of the working population, with a comparable rise in rate of older workers. However, despite the increasing presence of older workers, older people are underrepresented in the workforce. In the UK, by 2020, people over the age of 50 will make up almost half (47%) of the adult population, and one third (32%) of the available workforce [2]. Older people’s participation in the workforce declines with age; 81% of 50–54 year olds compared to just below 50% of 60–64 year olds participate in work [1, 3].

Internationally, in response to these demographic changes, there are a number of policies in place which seek to support older people to remain in work. Increasing the employment rates of older workers carries significant policy and economic potential, both through increasing taxable capacity but also reducing the economic strain on the economy via state benefits [4]. Often described as ‘active ageing’ policies [5, 6], these policies include measures such as increasing the statutory retirement age, and diminish early retirement incentives [7, 8]. The UK Government has taken similar steps to improve workplace retention of individuals aged over-50 (referred to in UK policy documents as ‘older workers’) [9]. This includes abolishing the compulsory age of retirement (enacted in the UK in 2011), and extending the period older workers are required to work before being eligible for the state pension. Historically in the UK, the state pension age (SPA) was 65 years for men, and 60 years for women. Under the Pensions Act 1995, SPA for women is to be equalised, and brought up to 65 years between 2010 and 2020. However the Pensions Act 2011 brought forward the time period this was to be actioned, and further raised pension age to 66 by 2020.

Despite these policies having a positive effect on the rates of work retention among older workers [10], these policies best help those who are currently in work and considering early retirement. Less is said in policy regarding ‘older workers’ who are currently unemployed and wish to return to employment.

Similar to other unemployed groups, over 50s experiencing unemployment are entitled to unemployment-related benefits from the UK Government. These benefits were Job Seekers Allowance (hereafter, JSA), and the health related benefit, Employment Support Allowance (hereafter, ESA), previously Incapacity Benefit (IB). Post 2013, individuals started to be assigned to a new benefit, Universal Credit (UC), which was introduced as a replacement to ESA and JSA as part of a wider reform of UK welfare policy. Most individuals claiming unemployment-related benefits are required to participate in work-related activity in order to receive payment (as per the current welfare-to-work policies). While there is not the space in this article to detail the extent of these policies, it is important to note that these policies have long been a feature of welfare policies in countries that have financial support available for those who are experiencing unemployment [11, 12]. Increasingly in these policies, receipt of benefit is conditional on individuals’ participation in work focused activities [13], in an attempt to “reactivate” this out of work population and encourage a return to work (the so called “labour market activation” policies) [14]. At the most basic level, to receive benefits, individuals are required to “sign on” at a Government job-centre, where they show evidence of job searching to an advisor. Alongside these interactions at the job-centre, or government funded service provider (as in the Work Programme), individuals may also be mandated to attend welfare-to-work support programmes.

From 1998 to 2009, the main source of welfare-to-work support for unemployed over-50s who were claiming unemployment benefits was a voluntary welfare-to-work programme, New Deal 50+ [15]. In 2011, this was replaced by the Work Programme (WP). Unlike its predecessor, the WP is mandatory to all ages, and offers support to all working age unemployed people who are in receipt of unemployment benefit (however, it is voluntary for those who have been assessed as having chronic health conditions that would limit the work they could do). The WP is a welfare-to-work initiative introduced by the then Conservative-Liberal Democrat Coalition Government in the UK. While WP providers are mainly from the private sector [16], it is overseen by the UK Government’s Department for Work and Pensions (DWP) [17].

Studies exploring what causes involuntary exit from the labour market have highlighted a variety of reasons. For example, poor self-perceived health is strongly associated with exit from paid employment among European workers [18, 19]; this is significant for the older working population as older workers are generally more likely than younger workers to have multiple chronic health problems, as multimorbidity (the presence of two or more associated chronic health conditions) and disability increases with age [20, 21]. However, it is not only poor-health that is associated with involuntary exit from the labour market. Other issues relate to redundancy, low skills, and care responsibilities which may push individuals out of work in order to care for sick partner or relatives [22–24]. Once they have exit the labour market, it is often difficult to return; the OECD describe early exit from the labour market as a ‘one-way street’ [1] and the UK charity Age UK refer to an ‘unemployment scrapheap’ for older jobseekers [25]. Some of these difficulties reflect the reasons for leaving, with poor health and issues managing health conditions highlighted as a major barrier to work [26, 27]. Older workers have more health conditions and those with more health conditions have a significantly low likelihood of being out of the labour force [28]. However, for those who do not have health conditions, they still face barriers in returning to work. For example, age discrimination from employers and recruiters, and skill gaps (which are often reported in ex-manual workers where IT skills were not a required part of their previous job) [29, 30]. Some of these barriers interact with have social class or gender [31], for example care commitments are more likely to be a barrier reported by women [32], while higher status, non-manual older workers are more likely to be in a position to negotiate flexible working or have it available to them, and are less likely to expect health to reduce their working lives [33]. Finally, there are issues regarding the local labour-market, particularly in depressed local economies or deindustrialised areas where job demand outstrips local supply [29, 34].

Long-term unemployment is seen to have a negative impact on health. While, formal retirement is seen as having positive impacts on both physical and mental health [35], similar to younger age groups, experience of unemployment is linked to negative outcomes in mental health and wellbeing [36, 37]. For older workers this may be more pronounced given the reduced opportunities for re-employment leading to lower social and mental engagement, lower control, and low self-esteem [38]. Given the difficulties faced by this group in RTW, and the associated health impacts of remaining unemployed [39], this is a group with important policy relevance [9].

In this paper, we explored the perceptions, attitudes, and experiences of RTW barriers among unemployed adults aged over 50 years. In doing so, we seek to answer the question how do age and health feature in the micro and macro-level factors in older unemployed workers lives, and how do these impact their discussions of returning to work?

## Methods

The qualitative data presented are part of a larger mixed-method longitudinal study involving collaboration between a team of academics and a major Work Programme (WP) provider. The aim of the overall study was to investigate the relationship between health, unemployment and the RTW process for the over-50s [40].

The collaboration enabled the academic team to access and analyse routinely collected data by the industry partner for the quantitative component, but also recruit individuals who were currently engaged in the WP for the qualitative component. To preserve confidentiality and anonymity of participants, and the freedom and ethical practice of the research team, the WP partners are unable to access audio recordings, view written transcripts of any interviews conducted, learn which of their clients participated in the study, or suggest changes to written reports.

### Recruitment and eligibility of study participants

Given the nature of the research collaboration, all participants were recruited from a private WP provider in Scotland [40]. Further criterion sampling, a purposeful sampling strategy, was used in this study. Participants were sampled based on age (over-50 years), duration of engagement with the WP (between three and 9 months), and location (based in Scotland). While engagement with the WP lasts 2 years, the research team sought to interview people in the early stages of the Work Programme with the hope to re-interview them after 12 months. There were no exclusionary criteria placed on gender, educational attainment, and time unemployed, to ensure we received a range of experiences.

As the WP is a UK Government intervention, the research team had to request access to contact details for WP clients from DWP. DWP required a two stage consent process. The first stage involved DWP writing to 900 eligible clients to request opt-in consent to share their information; the second stage involved the academic team writing to those individuals with additional information and to gauge their interest in participating. Of the 900 individuals who formed the potential cohort, 120 (13%) gave their initial consent to having their contact details shared. After receiving information relating to the study, 26 agreed to participate. The qualitative cohort was mostly representative of the wider quantitative cohort, with some differences in terms of health conditions, and age.

This is similar to other qualitative studies using criterion sampling [41], as recruitment was reliant on a cohort of individuals who were available and willing to share experiences. The 26 participants who were interviewed had with a range of health, work, skills, and educational experiences which enabled us to explore diversity of experience of unemployment in older populations.

### Interviews

The wider qualitative study involved two data collection time periods, one when participants were between 3 and 6 months in the WP (‘wave one’), and again when the participants were at the end of the intervention, between 16 and 24 months (wave two). The first interviews, of which the results of this paper are based, explore the participants’ experiences of unemployment prior to their experiences in the WP. While the first interview collected some data regarding participants’ early thoughts of the WP, the second interview explored WP in detail.

The wave one interview schedule (see Additional file 1) was developed from a literature review of previous studies exploring older adults’ experiences of unemployment and work, and an interest in the life-course approach [32, 42] to better understand the interaction between health and work over their working lives. Participants were asked questions based on previous experience of work (and reasons for leaving the workforce), how health changed over the life-course, experience of unemployment, and hopes for the future. Given the semi-structured nature of the interview, the interview schedule was also malleable to emerging themes. This was particularly important where participants spontaneously disclosed something that was not included in the interview schedule. Adaptions could then be made to ascertain whether the disclosure was the experience of one person, or it was a shared experience by others. Differences in terms of education level, gender, and location were noted and further explored further.

JN conducted all semi-structured interviews with participants in either their homes or in community locations, between November 2015 to July 2016. Participants were given an information sheet highlighting the voluntary and confidential nature of the study, prior to signing the consent form. The duration of interviews was between 35 min and two hours. Participants received a £15 gift voucher as an acknowledgement of their time, and were also recompensed for any out-of-pocket expenses they may have incurred in participation.

Interviews were recorded on an encrypted digital recorder and audio-files saved on an encrypted PC. Sound files were encrypted and sent using a secure file transfer protocol to be transcribed by a trusted third party company. Transcriptions were checked for quality purposes, and stored on a secure server.

### Analysis

A thematic analytical approach [43] was undertaken to identify the barriers and facilitators for participants in returning to work. At the start and during the analysis, JN listened to the audio and read transcriptions, taking notes on relevant points of interest. Coding of the data was led by JN, and performed using QSR NVivo 10© software. A sample of 25% of the transcripts was double-coded by SVK and HT to ensure validity and rigour [44]. From here, an initial descriptive coding framework was developed by JN, and following discussions with all authors and comparisons with the sub-analysis conducted by SVK and HT, a second order coding framework was developed which identified more conceptual themes and a wider narrative [45]. SVK and HT commented on the emerging findings and highlighted overlapping themes. In addition to seeking patterns within the data, particular attention was paid to contradictory data. This rigorous analysis technique ensured the findings presented were the product of discussion and agreement within the research team.

Analysis was also supported by the use of fieldnotes taken at time of interview, and a coding notebook that contained initial thoughts and feelings over the direction of the data.

## Results

In total, 26 participants currently engaged with the Work Programme were interviewed for this study. Key socio-demographic information for the participants is reported in Table 1. At the time of interview, two were employed and receiving ‘in work support’ from the Work Programme provider. ‘In work support’ is offered to any individual during the Work Programme who was able to return to work. The Work Programme advisor would stay in touch with the individual, offering support and advice where needed. The reason behind providing in work support is connected to the payment-by-results model of the Work Programme, where the company is paid primarily when individuals retain employment for an extended period of time. 24 of 26 participants were unemployed.

**Table 1:** Descriptive data

	Category	(*n* = 26)
Gender	Female	14
	Male	12
Age	50–54	13
	55–59	7
	60–64	6
Benefit type	Job Seekers Allowance	10
	Employment Support Allowance	14
	Universal Credit	2
Health condition	No	1
	Yes- mental health	7
	Yes- physical health	8
	Yes- both physical and mental health	10
Last occupation (based on standard occupational classification)	No employment history	1
	Elementary occupations	6
	Process/plant/machine operatives	3
	Sales and Service occupations	1
	Caring and leisure	5
	Skilled trades	2
	Administration/secretarial	1
	Associate professional and technical	4
	Professional occupations	3
	Senior officials	0
Educational attainment	No qualifications	6
	Standard grade/o-level/leaving cert	4
	A-levels/Highers	1
	Vocational qualifications (inc. city and guilds)	3
	College level qualification	6
	University degree	6
Length of unemployment	< 12 months	5
	1–2 years	0
	2–5 years	15
	> 5 years	6

The participants cited a range of individual and societal factors which impacted on their ability to return to work. Individual factors included health, skills, and education attainment, while wider societal factors included local economy, and the impact of changing welfare policy. Age was not reported as a standalone issue, but rather was seen to exacerbate a range of different issues. The interplay of age with individual and societal factors appeared to be central in older people’s discussions of barriers in RTW, both in terms of accessing and retaining employment. The remainder of this paper explores the interplay between age, individual, and societal factors in creating barriers to work. All names used are pseudonyms.

### Interplay between age and micro-level factors

Often, when participants discussed their current experience of unemployment, and their perceptions of returning to work, they cited micro-level factors which may play a role. This was especially seen with the interplay between health and age. As detailed in table one, the majority of participants had some sort of health condition, with many citing multiple conditions. Health was therefore seen as one of the main issues for participants. Some of the younger participants (aged 50–54 years) described feeling frustrated, as they felt young enough to return to work, but that their health was holding them back. Where participants had experienced a major health event (e.g. heart attack, major depressive episode), participants described exercising caution in their decision to return to work. Some described a worry of being pushed ‘*over the edge’* and imagined a situation where work-related stress (either physical or mental) may trigger another health event. In some cases, their health condition meant they would be unable to return to their previous job, and would therefore have to look for other work. For some, trying to find a post that would accommodate their health condition was difficult:

*I won’t be able to do anything in the past that I have done, you know? So I’m thinking about going, retraining. [I’m] struggling wi’, you know, the limits, the limitations on the sort o’ work that I’m qualified to do. I mean, I’m null an’ void, you know (Steve; condition: heart condition, knee replacement).*

There was a loss of identity for some participants when they found they would no longer be able to work in their previous jobs. Others described a loss of purpose. There were similar reports among those, typically men, who had developed chronic musculoskeletal problems which they attributed to previous long-term manual work, for example they reported difficulties lifting heavy loads, unable to sit for extended periods of time, or difficulties with dexterity. These health conditions prevented them from being able to return to their previous employer, but faced difficulties in searching for other posts:

They know you’ve got some illnesses. What you wanting me to dae? I cannae go and lift heavy things, I’ve got a weak heart. I’m no’ a brainy boy, didnae like school. I can count. I can read. I can write. But I’ll no’ understand it all. (Danny; condition: depression, skin condition, heart disease)

Some of these men described having low levels of literacy and numeracy, leaving school early or working in areas which did not require formal qualifications. Therefore, for these men, they faced several interrelated issues: low/no qualifications, a skill set specific to a particular trade, and health conditions that impact on their everyday behaviours. They discussed a worry that they would be seen as the ‘sick older worker’ rather than have a fair chance to show their abilities. This worry was often described as the need to be ‘*realistic*’ about their chances of returning to work. The need to be realistic was also felt by older participants (aged 60–64) who had chronic or fluctuating health conditions:

*I’ve only got 5 years left to work, and with my health…but being realistic, who would actually employ you? It’s not so much who would actually employ you, it’s…who would take into consideration, your health? (Janette; condition: scoliosis, arthritis in hip, depression).*

*I might look fine and for a few months I could probably work full time and then you get the day, or the couple of days where I’m that tried, I can’t do anything. So obviously, I can’t go to work. So then you’ve got time off. Perhaps a couple of months or whatever pass and you might get tired again so it’s more time off. (Liz; condition: osteoarthritis, bowel problems).*

These participants described difficulties not only with the symptoms of their conditions, but also with the associated treatment burden [46]. The treatment burden may involve adapted routines, multiple medications, trips to health professionals and making lifestyle changes. Participants who were likely to return to low-skill work voiced concerns over employers’ understanding of their healthcare needs.

### Interplay between age, policy, and work culture

It was not only micro-level factors such as health and skills that presented barriers to work, macro-level factors such as recruitment policies, retirement policy change and the demands of zero-hour contracts were also mentioned by participants. It appeared to suggest that while active ageing policies and the UK’s Fuller Working Lives strategy provided positive messages of encouragement towards working longer, these were tempered by discouraging experiences of ageism, employer desire for worker flexibility, and increasing care demands at home.

Ageism was discussed in two distinct ways by participants: direct and indirect. Discussions of direct ageism centred on experiencing negative reactions when disclosing their age at job interviews (e.g. through writing their date of birth on CVs or application forms). Experiences of indirect ageism were more subtle. These included requesting online submissions for jobs, which negatively impacted older people who were not confident in using computers, or asking for additional tasks to be completed prior to interview:

*When we went in, we’d to write a short essay about why you became a carer. And obviously I made a mess of it because I’ve never written an essay since I was at school and I didn’t know how to write the essay. “So I think that’s failed me in that as well”. (Marie; condition: anxiety, arthritis, pancreatitis).*

Marie had been a full-time-carer for a sick relative for 10-years; after they died, Marie was interested in returning to work, and transforming her personal experiences into a formal qualification. As part of her application to the local college, she was required to complete a reflective essay describing her interest in the qualification. This was a standard requirement, and while it did not directly discriminate against any applicant, Marie noted she was placed at a disadvantage compared with a recent school-lever as she had not written an essay in 35 years.

Concerns about fitting into the new flexible job market were also discussed in this group. While some participants discussed the importance of balancing their health needs with the demands of a flexible job market; others suggested the flexibility of the job market itself, with the prevalence of short-term, zero-hours, and seasonal work, providing cause for concern. Compared to their previous experience of long-term and financially stable jobs in their jobs in their earlier life, these flexible contracts were framed as a financial risk, with the changeable hours in zero-hours posts meaning there was a risk that participants would not be able to pay bills at the end of the month. For participants living in smaller deindustrialised towns, they described being in a difficult situation. They described living in a depressed local economy, with a highly competitive job market for the few posts that were available. One option was to travel to the nearest large city. However this also had its difficulties:

*I did get an interview with [fast food chain] once…it was a zero hour contract but …they wouldn’t guarantee any hours and then she really wanted somebody that could come in any of these hours… they want you to be there from half five in a morning to after midnight…I couldn’t get there for half five, or get back after midnight. (Beth; condition: depression, rheumatoid arthritis).*

Unlike large cities, the public transport in rural areas was poorer, with buses often ceasing service after 9 pm. This meant that the demands of zero-hours contracts were difficult to achieve for individuals who could not drive as they would not be able to be available for very early or very late shifts. Given the considerable distance between Beth’s home and the fast food location, she could also not rely on taxis, as the fare would deduct a significant amount of money from her wage.

Participants also described the interaction between meso and macro level factors. One such issue was raised by older women participants (aged 60–64). For them, discussions of returning to work were enmeshed in discussions of the recent changes to the pension age. At the time of fieldwork, there had been an announcement where pensionable ages of men and women had been equalised. The women affected by this policy change described feelings of anger and frustration that their plans for the future had been affected by these changes, particularly where they had experienced poor health and expected to be able to concentrate on their health over the next year. Others described impact of the pension change on the conflicting demands of grandparenthood and searching for work:

*I would rather help my daughter and watch my grandson, and let her get a job than actually have a job myself. I would rather say “she’s young, why not get a wee job or career for her” But then they don’t want that. It’s another thing to make older people sign on for longer than to work longer. It’ll be harder for grandparents to help younger people with the family, they’ll say “no I’ve got to sign on, I can’t help you watch the weans [children]” (Beth; condition: depression, rheumatoid arthritis).*

Demands of the family, and their expectation of being able to take an active role in grandparenting to lessen the burden on their children, were in conflict with the demands placed on them by the jobcentre. A failure to “sign on” at the job centre would risk individuals receiving a financial sanction on their benefit payment, which would negatively impact her ability to pay rent, food bills, and utilities.

It appeared for participants to be successful in returning to work, they had to be in receipt of a range of factors. These included being relatively young (50–54 years), be able to work computers to search for jobs, live in an area with a healthy local economy, have some idea of where they would be able to work, and be able to manage their health condition:

*I can cope with, you know, my leaking bladder and what-have-you. I can do that. But I couldn’t have coped with it if I had to go in for more surgery. I just can’t do that. So I can’t do kind of manual lifting so I couldn’t go back to do kind of, you know, healthcare… I actually go and help out at a wee [little] café on a Saturday and Sunday. Its minimum wage and it’s all young school kids and me, but it’s really healthy, I have fun and I’m still part of the workforce (Marianne; condition: depression, prolapse).*

After a period of major depression, and physical health complications after surgery, Marianne found she was not able to RTW in her previous capacity. She described problems with self-confidence and a loss of direction, and also being cautious regarding her physical capabilities. However, after talking with a friend she met in a local coffee shop, she took on a part time post which offered a physically low-impact job, in a customer facing environment. Due to the existing relationship, she felt her health conditions were understood, and has flexible working conditions.

## Discussion

Employment of the over-50s has become a significant area of policy in both the UK and other OECD countries. However, the significant decrease in employment and the ‘one-way-street’ of unemployment has created interest into how best to support this group. This study explored the perceptions and experiences of returning to work with a group of unemployed older workers who were among the furthest away from the labour market. Specifically, we sought to answer the question of how age and health feature in the micro and macro-level factors in older unemployed workers lives, and how these could be seen in their narratives of returning to work.

Similar to previous studies, age was never seen as a sole cause of their difficulties in returning to work. Instead, their difficulties were more complex, and could be viewed as the product of the interplay between age and micro and macro-level issues [30, 47, 48]. This can be seen when participants’ issues of health, care commitments, and skill levels were compounded by wider issues such as poor recruitment strategies, depressed local labour demand, and precarious employment. These issues mirror discussions of ‘supply’ and ‘demand’ from the employability literature [49]. From the ‘supply’ side, we see examples such as older ex-manual workers (mostly men) whose health condition prevents them from returning to a similar role, voice concerns about their lack of transferable skills in looking for a new job. For some of these participants, lack of skills included numeracy, IT literacy, as well as employability skills such as interview skills or how to write CVs. From the ‘demand’ side, there were concerns over low levels of “decent” job opportunities in more rural locations, and the prevalence of zero-hour contracts. Zero-hours contracts were often discussed by participants who had money concerns as they associated the unpredictability of zero-hours with worries about not being able to pay bills at the end of the month, which deterred some participants from applying to jobs [50, 51].

We have also attempted to locate these findings within wider policy developments such as active ageing policies (specifically the extension of women’s working age), and labour activation policies. From the findings, we see the negative impact these macro-level policies have on individuals, with older women discussing the frustration at not being able to take more of a caring role for grandchildren for fear of being financially sanctioned for not attending appointments or seeking enough work. The findings of this study also highlight an issue of (in)flexibility, both at the micro and macro-level. For over-50s who are able to re-join the labour market after a period of unemployment, the modern world of employment they are re-entering often demands a great deal of flexibility from its workers: variable shift patterns, being available on demand, and being able to fulfil different roles [50, 51]. This demand for flexibility can itself be read as an *inflexible* policy, with the return to work prospects of those who cannot adhere to the hyper-flexibility demands of the labour market, reduced. This was particularly discussed by participants who struggled with the treatment burden associated with their chronic health condition. Their capacity to return to work full-time, or take on zero-hours shift work appeared diminished by the differing burdens associated with their condition, and their need for a flexible workforce. Their needs included an ability to plan for GP or health-practitioner appointments (which may impact negatively on their ability to attend work), modified environments (to enable them to work safely, particularly where there were MSK related issues), taking medications (which may impact on cognitive functioning), but also finding a supportive employer who would understand and accommodate these needs [52, 53]. A similar need for flexibility was also discussed by individuals with care commitments, where their availability was dependent on the needs of others. Care commitments also had a gendered component [32], as more female than male participants discussed the desire to take a more active grandparenting role which therefore required a rebalance in the conflicting demands of home and work.

Acknowledging these interactions at the micro and macro-level, and the issues pertaining to (in)flexible demands of the economy, the unemployment experienced by the participants was not divided neatly into those who can and cannot work. Many of the participants in this study expressed an interest in returning to work, and commented that they were actively searching for employment opportunities, but they felt that they required work to fit into their own demands: of taking time off to cope with health conditions, of taking care of grandchildren, of receiving additional training, and of having their own ‘real world’ experiences given appropriate credit alongside academic accomplishments. This group may reside in a ‘grey area’, comprising of individuals who may experience limitations on what they could do, yet are capable to participating in the workforce in some way [31, 54] but require further assistance to RTW.

### Policy implications

While employability ‘active ageing’ policies [6, 10] encourage the retention of older workers in employment [6, 9, 10], based on the findings of this study, we suggest that more should be done to support those who have fallen out of the labour market. The participants interviewed for this study were among the furthest away from the labour market and experienced multiple barriers to work. Supporting these individuals to return to work may require a multi-faceted approach, encompassing healthcare, skills and training, and local employers. Making visible the support available for individuals with health care requirements, such as the UK Government’s Access to Work grants which help pay for special equipment, adaptions, or a support worker to assist with work tasks [55]. This may help the confidence of unemployed people to apply for jobs, but also of employers to support those with health conditions. Also, having information about this support available at employability services, and train advisors on how to signpost individuals to these services.

However, improving return to work for older workers with disabilities, or chronic health conditions, would also require employers to observe the ‘disabling’ factors of work: attitudes of staff towards disability, clarifying employers’ flexible working policy for those with chronic health conditions, and promote the recruitment of older workers with health conditions into the workforce. While the UK has some apprenticeships for over-50s, these are often based in large companies, and target individuals with high academic levels. We suggest this policy should also focus on those over-50s who, like the participants in this study, have found they are unable to do their long-term job and need assistance in transferring their skills to a new post. In addition, given the anxiety of some participants towards the prevalence of precarious contracts whose unpredictable hours may negatively impact on those with mental health conditions, more should be done to create more secure job contracts.

Finally, policy makers should also explore and reward alternative positive destinations for older people where paid employment may be more difficult to obtain, for example voluntary work. A failure to do so may simply lead to an extension to the time people remain unemployed before they are able to qualify for state pension.

### Strengths and limitations

This study elicited views from participants with varying degrees of health needs, and with varying experiences of unemployment. The variation of experience achieved in this study is one of the key strengths. Our study has international policy relevance as low income, older working age people in receipt of unemployment benefits are an important target population in many countries. The findings of this study are specifically relevant for those countries that follow a similar neoliberal approach to unemployment policy, with an emphasis on labour market activation (such as USA, Canada, and Australia). Another key strength is the ability to work with a Work Programme provider, which enabled us to access to over-50s participating in this intervention. However, given the mandatory nature of the Work Programme, this may also have acted against us, as people may have been unwilling to participate for fear that we would report their perceptions to their advisor. This was an issue that was experienced during the interviews, as participants required further reassurance of anonymization and confidentiality. We acknowledge that in gathering narratives from participants with varying degrees of health needs, we may be missing the experiences of over-50s who did not have health conditions; however this reflects the participants who agreed to participate in the study.

## Conclusion

This study elicited views from participants with varying degrees of health needs, and with varying experiences of unemployment. We note that wider macro-level policy changes, such as changes to retirement age, and changes in the local economy, had an impact on participants’ discussions of returning to work. These discussions were informed by the participants’ experience of health, and health management, but also their experience of skills and training. While age was not seen as an issue in and of itself, it was an important factor which influenced other ongoing concerns in their lives.

## Additional file

Additional file 1: Interview schedule. (DOCX 12 kb)

## Abbreviations

DWP: Department for Work and Pensions
ESA: Employment Support Allowance
JSA: Job Seekers Allowance
MSK: musculoskeletal
OECD: Organisation for Economic Co-operation and Development
RTW: Return to Work
UK: United Kingdom
WP: Work Programme
