# Supplementary material for: Role of age and health in perceptions of returning to work: a qualitative study
Source: BMC Public Health. 2019 May 2;19:496. doi: 10.1186/s12889-019-6819-9 (PMC6498557; doi:10.1186/s12889-019-6819-9)
Supplement: Supplementary file 1 — Interview schedule. (DOCX 12 kb) [file 12889_2019_6819_MOESM1_ESM.docx]

How would you describe your physical health at the moment?

How would you describe your mental health?

Do you have any specific medical conditions?

Do you think your health just now has an effect on a) your ability to socialise b) your daily routine c) your search for work?

What medications are you currently taking?

Do you see a doctor, GP, other medical professional?

Have they ever spoken to you about working?

Have you had experience of having your health assessed for the purpose of benefit entitlement?

What benefit are you currently claiming? How long have you been in receipt of this?

Do you receive any other financial support? (Eg PIP, housing benefit etc)

What was your last experience of paid employment? Where was it, how long were you there, what did you have to do in the job? How did you learn about this job?

When did you leave this job? What were the reasons behind this?

When you look back at your working life, would you say the majority has been employed, unemployed, caring for family, or a combination of the above?

Have you ever considered volunteering?

Do you think health and work are related? In what ways?

Have you experienced any positive or negative attitudes by employers to people over-50?

Do you think unemployed people who are over-50 would want to return to work?

What do you think are the main challenges of finding work for people over 50?

Do you have any concerns about returning to work?
